# Association between RC/HDL-C ratio and risk of non-alcoholic fatty liver disease in the United States

**DOI:** 10.3389/fmed.2024.1427138

**Published:** 2024-07-29

**Authors:** Yanyan Xuan, Weike Hu, Yudan Wang, Jingwen Li, Lisha Yang, Songping Yu, Dongdong Zhou

**Affiliations:** ^1^Department of Hospital Infection, The First Affiliated Hospital of Ningbo University, Ningbo, Zhejiang, China; ^2^Department of Hepatology, The First Affiliated Hospital of Ningbo University, Ningbo, Zhejiang, China; ^3^Department of Emergency, The First Affiliated Hospital of Ningbo University, Ningbo, Zhejiang, China; ^4^Department of Intensive Care Unit, The First Affiliated Hospital of Ningbo University, Ningbo, Zhejiang, China; ^5^Department of Geriatrics Medicine, The First Affiliated Hospital of Ningbo University, Ningbo, Zhejiang, China; ^6^Department of General Medicine, The First Affiliated Hospital of Ningbo University, Ningbo, Zhejiang, China

**Keywords:** remnant cholesterol, high-density lipoprotein cholesterol, NAFLD, NHANES, steatosis

## Abstract

**Background:**

The occurrence of non-alcoholic fatty liver disease (NAFLD) is increasing worldwide. The link between serum remnant cholesterol (RC) to high-density lipoprotein cholesterol (HDL-C) ratio and NAFLD remains unclear. Therefore, we sought to clarify the relationship between the RC/HDL-C ratio and the NAFLD.

**Methods:**

Data for our cross-sectional study came from the 2017–2018 National Health and Nutrition Examination Survey (NHANES) with 2,269 participants. Associations between RC/HDL-C levels and the prevalence of NAFLD and hepatic fibrosis were evaluated using adjusted multivariate logistic regression analyses. A generalized additive model examined the non-linear relationship between RC/HDL-C and the probability of developing NAFLD.

**Results:**

Among 2,269 participants, 893 (39.36%) were diagnosed with NAFLD. In each of the three models, RC/HDL-C and NAFLD had a strong positive statistical relationship: model 1 (OR = 9.294, 95%CI: 6.785, 12.731), model 2 (OR = 7.450, 95%CI: 5.401, 10.278), and model 3 (OR = 2.734, 95%CI: 1.895, 3.944). In addition, the subgroup analysis by gender and BMI suggested that RC/HDL-C showed a positive correlation with NAFLD. The RC/HDL-C ratio was positively correlated with the degree of liver steatosis. There was an inverted U-shaped connection between the prevalence of NAFLD and RC/HDL-C, with an inflection point of 0.619 for all participants and 0.690 for men. Receiver operating characteristic (ROC) analysis showed that the predictive value of RC/HDL-C for NAFLD (area under the curve: 0.7139; 95%CI: 0.6923, 0.7354; *P* < 0.001), was better than traditional lipid parameters.

**Conclusion:**

Increased RC/HDL-C levels are independently associated with an increased risk of NAFLD and the severity of liver steatosis in the American population. In addition, the RC/HDL-C ratio can be used as a simple and effective non-invasive biomarker to identify individuals with a high risk of NAFLD.

## Introduction

The chronic metabolic disease known as non-alcoholic fatty liver disease (NAFLD) is typified by the buildup of lipid deposits in hepatocytes without any apparent etiology or history of alcohol misuse (1). Over 600 million people globally are predicted to have NAFLD, with over 400 million of those suffering in Europe and the US (2). The prevalence of NAFLD is estimated to exceed 30% in many countries (3). NAFLD has emerged as a significant contributor to the incidence of liver transplantation in the United States. Consequently, NAFLD represents a global public health problem that cannot be ignored. The development of NAFLD is primarily associated with metabolic factors, including the consumption of a high-fat diet, a sedentary lifestyle, and the presence of metabolic syndromes such as obesity, hyperlipidemia, hypertension, and Type 2 Diabetes (T2DM) (4). Patients with NAFLD exhibit significant dysfunction of hepatic fat metabolism, resulting in the accumulation of large amounts of fat-like substances in hepatocytes, which progresses from simple non-alcoholic fatty liver (NAFL) to non-alcoholic steatohepatitis (NASH) and eventually develops into liver fibrosis, liver cirrhosis, end-stage liver disease, and even liver tumor (5). Consequently, implementing effective prevention, diagnosis, and evaluation strategies for NAFLD is paramount, particularly concerning developing non-invasive diagnostic techniques that significantly impact patient follow-up and monitoring.

RC is a new non-traditional blood lipid index proposed in recent years that refers to the total cholesterol rich in triacylglycerol-rich lipoproteins (TRLs), which is formed when TRLs are cleared of triglyceride (TG) by lipoprotein lipase (LPL). Under fasting conditions, RC is composed of VLDL cholesterol (VLDL-C) and medium-density lipoprotein cholesterol (IDL-C), and in the non-fasting state, RC is composed of remnants of chylomicrons (CM) (6). At present, it has been found that one-third of total plasma cholesterol is RC, and the increase of RC is associated with a variety of cardiovascular diseases, such as vascular endothelial dysfunction, increased carotid intima thickness, and left ventricular diastolic dysfunction (7). Insulin resistance is a condition in which, for a variety of reasons, insulin promotes glucose uptake and utilization, preventing plasma glucose from being maintained at normal levels, leading to increased lipid synthesis, impaired inhibition of fatty acid catabolism, and excessive accumulation of triglycerides in the liver, which is the leading cause of NAFLD (8). NAFLD and insulin resistance co-exist, and the associated factors that cause insulin resistance also affect NAFLD. Insulin can increase lipase activity, which increases triglyceride uptake by adipose tissue, promoting fat storage in the liver, and leading to NAFLD and excessive lipid deposition, further exacerbating insulin resistance (9). The main risk factor for NAFLD is abnormal lipid metabolism, which is manifested by a decrease in HDL-C. HDL-C has antioxidant properties and an enhanced cholesterol-scavenging capacity. HDL-C lowers serum cholesterol levels by reversing the cholesterol transport system and facilitating the removal of cholesterol from the diet, which plays a vital role in the occurrence and development of NAFLD (10).

Since the ratio of lipids to lipoproteins can indicate interactions between lipid components, elevated RC/HDL-C is associated with the occurrence and development of cardiovascular disease, cerebrovascular disease, and T2DM (11–13). Abnormal lipid metabolism is a hallmark of NAFLD, and NAFLD is closely associated with metabolic disorders such as T2DM. However, little research has been done on the connection between increased RC/HDL-C and the risk of NAFLD, particularly in the general U.S. population. In light of this, we carried out the following cross-sectional study using pertinent data from the 2017–2018 National Health and Nutrition Examination Survey (NHANES) to determine the association between RC/HDLC and NAFLD status as well as the progression of hepatic steatosis and fibrosis in the general population of the United States.

## Methods

### Study design and population

The present research is a cross-sectional, retrospective analysis of secondary data that was taken from the 2017–2018 NHANES database, where liver ultrasonography Transient Elastography (TE) examinations were conducted. The NHANES, a biennial nationally representative survey of the country's population, was carried out by the CDC's National Center for Health Statistics (NCHS). Since its inception in 1999, the NHANES has been performed annually, assessing around 5,000 participants. The NHANES survey uses a complex, multi-stage design to facilitate the collection and analysis of nationally representative data designed to assess the health and nutritional status of noninstitutionalized people throughout the United States. A thorough assessment procedure is carried out on the participants, which includes a home interview and an examination at a mobile examination center (MEC) that entails laboratory testing, specialized measures, and physical examination. Every participant completed a written consent form, and the NCHS Research Ethics Review Board approved the NHANES study protocol.

**Figure 1:**
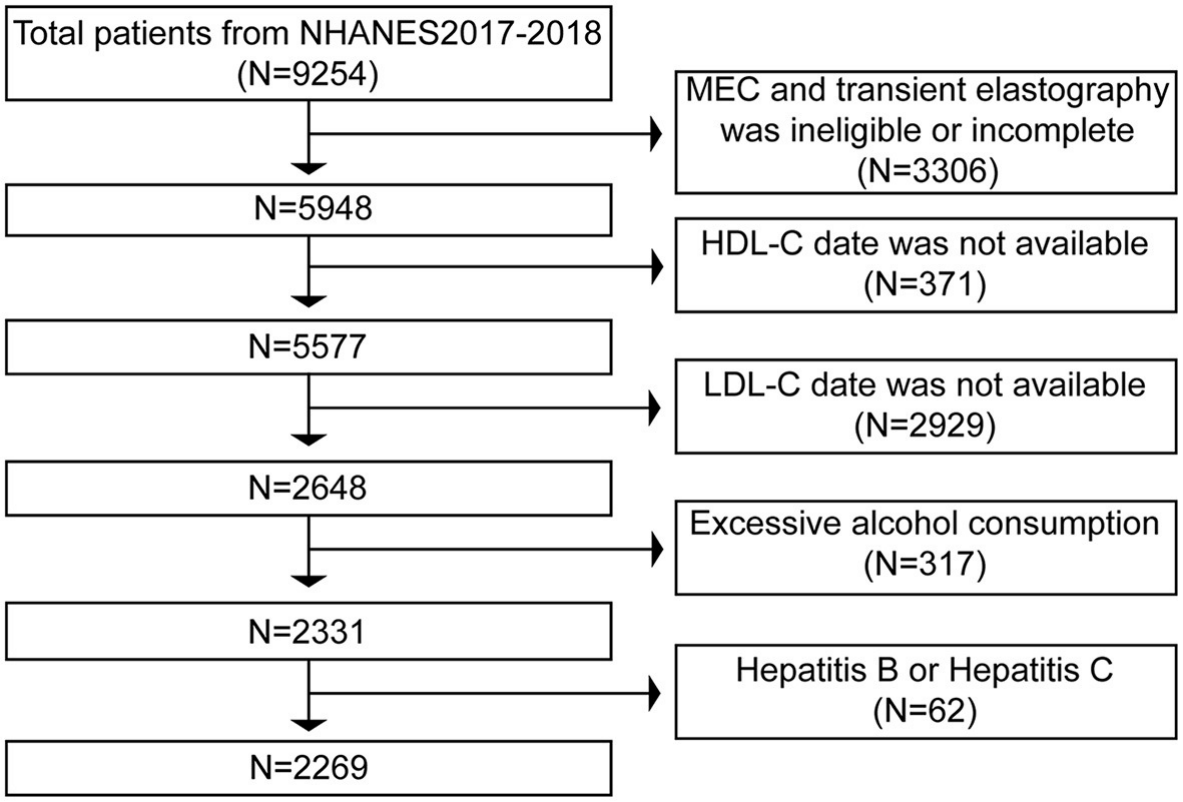
Flowchart of the study participants.

The primary foundation for our study's exclusion criteria was primarily based on the diagnosis of NAFLD. 9,254 individuals completed the poll in total between 2017 and 2018. First, 3,306 patients were excluded from the analysis, comprising those with incomplete MEC examinations and those without TE results. Next, we removed the 2,929 people for whom LDL-C data were unavailable and the 371 for whom HDL-C values were unavailable. Finally, we excluded 317 participants who consume excessive amounts of alcohol (defined as males > 21 standard drinks per week and females > 14 standard drinks per week), as well as 31 people who have hepatitis B and 31 people who have hepatitis C. Ultimately, 2,269 people were analyzed using these exclusion criteria (Figure 1).

### Definition of NAFLD

A controlled attenuation parameter (CAP) in vibration-activated transient elastography (VCTE) has been used to identify NAFLD. Higher readings indicate increased liver fat content. We were founded on data from an earlier population-based meta-analysis evaluating the disease's CAP diagnostic cutoffs. NAFLD was diagnosed using a CAP score of at least 274 dB/m (14).

### Vibration controlled transient elastography (VCTE)

In most cases, a liver biopsy is the most accurate approach for identifying fatty liver steatosis and liver fibrosis and diagnosing NAFLD. But in addition to being expensive and having little repeatability, the procedure has been associated with potential complications, including bleeding, infection, or even death. In the last several years, VCTE has become the most widely used non-invasive and economical technique for precisely evaluating liver fibrosis and steatosis in fatty liver disease (15). The CAP and liver stiffness measurement (LSM) are two well-validated, non-invasive methods that VCTE may use to detect the presence of liver steatosis and fibrosis.

Throughout the 2017–2018 cycle, VCTE was detected using FibroScan^®^ model 502 V2 Touch via medium (M) and extra-large (XL) probes. After 2 days of instruction with a skilled technician, NHANES technicians conducted the test. Based on available clinical data, the LSM values and CAP levels positively correlate with liver fibrosis and the degree of hepatic steatosis. Tests were considered credible only if LSM results of >10 interquartile range (IQR)/median <30% were obtained after a minimum fasting period of at least 3 h. According to an earlier study, the cutoff value of hepatic steatosis determined by VCTE using CAP was ≥ 274 dB/m (14). Furthermore, significant liver fibrosis (F2), advanced liver fibrosis (F3), and cirrhosis (F4) are indicated by LSM values of 8.0, 9.7, and 13.7 kPa, respectively (16).

### Variables

The formula of RC was total cholesterol (TC) less HDL-C and low-density lipoprotein cholesterol (LDL-C) (17). The RC/HDL-C was computed by dividing the RC by the HDL-C. Before beginning the laboratory examination, every subject fasted for over 9 h. An auto analyzer for venous blood samples was used to measure the following parameters: platelets (PLT), C-reactive protein (CRP), glucose, glycosylated hemoglobin (HbA1c), serum lipids, liver enzymes, and fast glucose. The level of liver fibrosis, the degree of hepatic steatosis, and the prevalence of NAFLD and liver fibrosis were regarded as dependent factors, whereas RC/HDL-C was the independent variable. This analysis used the established confounders from earlier studies and clinical practice as covariates. In our investigation, the categories utilized as covariates were gender, race, smoking status, hypertension status, and diabetes status. The subsequent continuous variables were also used as covariates in our analysis: CRP, PLT, liver enzymes, SUA, serum lipids, Body mass index (BMI), fast glucose, fast insulin, HbA1c, and systolic/diastolic blood pressure (S/DBP).

T2DM was diagnosed using the following criteria: First, self-reported diabetes; second, use of anti-diabetic medications; third, measurement of fasting plasma glucose (FPG) of 126 mg/dl (7 mmol/L) or higher; fourth, measurement of random plasma glucose of ≥200 mg/dl (11.1 mmol/L); and fifth, measurement of HbA1c of ≥ 6.5% (18). If any of the following criteria were present, hypertension was defined as (1) Measured systolic blood pressure of more than 140 mmHg or diastolic blood pressure of more than 90 mmHg; (2) Anti-hypertensive drugs are being used at the moment; (3) Self-reported hypertension (16). The formula for calculating BMI was to divide the weight in kilograms by the height in square meters (19).

### Statistical analysis

The data was analyzed using EmpowerStats software (version 4. https://www.empowerstats.com) and Package R version 3.4.3. The data analysis used the proper weighting that the NCHS suggested to guarantee that the results appropriately reflect the US population. To be considered statistically significant, a two-tailed *P* value has to be <0.05. If continuous variables were not normally distributed, their values were presented as medians or interquartile ranges (IQRs); if they were, the data were expressed using weighted means. Categorical variables have been described using frequencies and proportions. Three types of logistic regression models were created once RC/HDL-C was categorized quarterly to investigate the association between RC/HDL-C and NAFLD as well as liver fibrosis: (1) No covariates have been adjusted for, (2) age, gender, and race adjustments have been made, (3) all pertinent variables, including age, gender, race, LSM, DBP, SBP, hypertension, BMI, T2DM, smoke, HBA1C, CRP, PLT, fast glucose, fast insulin, total cholesterol (TC), liver enzymes, total bilirubin (TBIL), physical activity level, statin use, and SUA, have been adjusted for.

Additionally, we utilized classified multivariate logistic regression to carry out subgroup stratification by gender or BMI analysis to find appropriate groups. Furthermore, smooth curve fitting and threshold effect analysis were used to analyze further the non-linear relationship between the RC/HDL-C ratio and NAFLD. When nonlinearities were identified, binary linear regression models were run on both sides of the threshold point. To evaluate the efficacy of RC/HDL-C or other biochemical measures in detecting NAFLD, ROC curve studies were conducted. The goal was to determine the ideal ratio of RC/HDL-C to estimate the risk of NAFLD in this group. By maximizing the Yoden index, optimal cutoffs were determined. When a *p*-value of <0.05 was reached, the result was deemed statistically significant.

## Results

### Demographic and clinical characteristics of the study population

A total of 2,269 individuals participated in the study. The mean age of the participants was 42.78 ± 19.11 years, with 52.83% identifying as female and 47.17% as male. The weighted distributions of the characteristics according to whether or not they were NAFLD are shown in Table 1.

**Table 1:** Weighted characteristics of participants with and without NAFLD status.

**Characteristics**	**Non-NAFLD (*n* = 1,376)**	**NAFLD (*n* = 893)**	***P* value**
Age (years)	38.99 ± 19.26	48.84 ± 17.20	<0.0001
Age (%)			<0.0001
<20	18.32	5.01	
≥20, <40	38.29	27.03	
≥40, <60	25.81	38.14	
≥60	17.58	29.82	
Gender (%)			0.0066
Male	44.92	51.78	
Female	55.08	49.23	
Race (%)			0.0011
Mexican American	8.89	14.04	
Other Hispanic	7.76	5.87	
Non-Hispanic White	60.06	60.59	
Non-Hispanic Black	11.96	9.31	
Non-Hispanic Asian	6.26	6.05	
Other race	5.07	4.14	
Smoking behavior (%)			0.0394
Never smoke	68.23	63.06	
Ever smoke	20.98	24.11	
Current smoke	10.79	12.82	
Hypertension (%)			<0.0001
No	74.96	45.78	
Yes	25.04	54.22	
T2DM			<0.0001
No	93.61	77.31	
Yes	6.39	22.69	
Physical activity level			0.0795
Inactive	48.35	47.87	
Moderate	8.05	10.90	
Active	43.61	41.23	
BMI (kg/m^2^)	25.93 ± 5.68	33.33 ± 7.22	<0.0001
BMI (%)			<0.0001
<25	49.02	7.63	
≥25, <30	32.09	28.29	
≥30	18.89	64.07	
Waist circumference (cm)	89.97 ± 14.48	109.59 ± 15.51	<0.0001
Hip circumference (cm)	101.08 ± 11.50	114.08 ± 14.19	<0.0001
SBP	117.41 ± 17.31	126.12 ± 16.72	<0.0001
DBP	69.74 ± 12.18	73.35 ± 12.14	<0.0001
PLT (10^9^/L)	239.90 ± 58.35	245.49 ± 59.68	<0.0280
CRP (mg/L)	2.91 ± 7.30	4.56 ± 6.97	<0.0001
Fast glucose (mmol/L)	5.23 ± 1.03	6.07 ± 1.86	<0.0001
Fast insulin (mIU/L)	9.41 ± 8.14	17.65 ± 14.92	<0.0001
Glycohemoglobin (%)	5.41 ± 0.63	5.87 ± 0.99	<0.0001
ALT (IU/L)	18.42 ± 13.93	26.27 ± 18.68	<0.0001
GGT (IU/L)	20.88 ± 21.36	32.14 ± 35.59	<0.0001
AST (IU/L)	20.33 ± 10.58	22.48 ± 12.59	<0.0001
ALP (IU/L)	85.85 ± 55.32	80.64 ± 30.54	0.0109
TBIL (mg/dL)	0.52 ± 0.33	0.47 ± 0.25	<0.0001
TG (mg/dL)	100.66 ± 53.37	141.48 ± 70.23	<0.0001
TC (mg/dL)	178.56 ± 38.32	187.53 ± 41.41	<0.0001
HDL-C (mg/dL)	56.48 ± 14.35	50.43 ± 14.69	<0.0001
LDL-C (mg/dL)	104.91 ± 33.48	112.10 ± 36.23	<0.0001
SUA (mg/dL)	5.01 ± 1.32	5.71 ± 1.41	<0.0001
RC (mg/dL)	17.17 ± 10.40	25.01 ± 13.58	<0.0001
RC/HDL-C	0.34 ± 0.28	0.56 ± 0.40	<0.0001
CAP (dB/m)	218.05 ± 36.92	323.13 ± 36.80	<0.0001
LSM (kPa)	4.85 ± 2.72	6.54 ± 5.08	<0.0001

Mean ± SD was for continuous variables. A weighted linear regression model for categorical variables calculated the p-value. The weighted chi-square test calculated the p-value.

In addition, subjects with NAFLD were more likely to be older, men, and Mexican American, had higher incidences of hypertension and T2DM, as well as smoking. Much higher S/DBP, BMI, waist circumference, hip circumference, LSM, CAP, triglycerides (TG), fast glucose, TC, fast insulin, low-density lipoprotein cholesterol (LDL-C), HbA1c, alanine aminotransferase (ALT), PLT, aspartate aminotransferase (AST), CRP, gamma-glutamyl transpeptidase (GGT), SUA, alkaline phosphatase (ALP), conversely, HDL-C and TBIL values were significantly lower in individuals with NAFLD (*P* < 0.001 for each). Further, the RC/HDL-C of the non-NAFLD group was considerably lower than that of the NAFLD subgroup (0.34 ± 0.28 vs. 0.56 ± 0.40, *P* < 0.0001). Nonetheless, there were no discernible differences in the amounts of physical exercise.

### Correlation between NAFLD and RC/HDL-C

The association between NAFLD prevalence and RC/HDL-C ratios was investigated using multiple regression analysis in Table 2. Positive associations were found between RC/HDL-C and the risk of NAFLD in all three of the multivariable logistic regression models: model 1 (OR = 9.294, 95% CI: 6.785, 12.731), model 2 (OR = 7.450, 95% CI: 5.401, 10.278), and model 3 (OR = 2.734, 95% CI: 1.895, 3.944). Moreover, individuals in groups 3 and 4 showed increases in NAFLD risks of 59.0 and 156.6%, respectively, compared to the lowest ratio of RC/HDL-C (Q1) in model 3 (*P* for trend <0.05). The results showed that those with greater RC/HDL-C than those with lower RC/HDL-C were more likely to develop NAFLD.

**Table 2:** Associations between remnant cholesterol to high-density lipoprotein cholesterol ratio (RC/HDL-C) and NAFLD status.

	**Model 1 *OR* (95% CI), *P* value**	**Model 2 *OR* (95% CI), *P* value**	**Model 3 *OR* (95% CI), *P* value**
**RC/HDL-C**	9.294 (6.785, 12.731) <0.001	7.450 (5.401, 10.278) <0.001	2.734 (1.895, 3.944) <0.001
Q1 (0.026–0.191)	Reference	Reference	Reference
Q2 (0.192–0.319)	2.125 (1.606, 2.811) <0.001	2.000 (1.501, 2.663) <0.001	1.247 (0.861, 1.806) 0.24346
Q3 (0.320–0.538)	3.763 (2.866, 4.940) <0.001	3.145 (2.371, 4.172) <0.001	1.590 (1.104, 2.289) 0.01268
Q4 (0.540–3.292)	8.229 (6.248, 10.839) <0.001	6.897 (5.161, 9.217) <0.001	2.566 (1.757, 3.749) <0.001
*P* for trend	<0.001	<0.001	<0.001
**Subgroup analysis stratified by sex**			
Men	7.593 (4.985, 11.565) <0.001	6.547(4.251, 10.084) <0.001	2.613 (1.582, 4.316) <0.001
Women	11.300(7.034,18.153) <0.001	8.714(5.388, 14.091) <0.001	2.776 (1.595, 4.831) <0.001
**Subgroup analysis stratified by BMI**			
<25	11.776(5.762, 24.067) <0.001	6.629 (3.048, 14.417) <0.001	5.383(1.974, 4.680) 0.001
≥25, <30	4.041 (2.445, 6.680) <0.001	3.387 (2.025, 5.666) <0.001	2.944 (1.601, 5.416) <0.001
≥30	5.299 (3.833, 7.325) <0.001	3.273 (1.960, 5.465) <0.001	2.154 (1.234, 3.760) 0.007

Model 1: no covariates were adjusted. Model 2: age, gender, and race were adjusted. Model 3:age, gender, race, hypertension, BMI, T2DM, smoke, LSM, DBP, SBP, HBA1C, CRP, PLT, fast glucose, fast insulin, ALT, AST, GGT, TC, TBIL, physical activity level, statin use, and SUA were adjusted. In the subgroup analysis for sex, the model was not adjusted for sex, and in the subgroup analysis for age, the model was not adjusted for age.

All three models' subgroup analyses, which were gender-stratified, revealed a positive relationship between male NAFLD risk and RC/HDL-C in model 1 (OR = 7.593, 95% CI: 4.985, 11.565), model 2 (OR = 6.547, 95% CI: 4.251, 10.084), and model 3 (OR = 2.613, 95% CI: 1.582, 4.316). For females, we likewise found that all three models showed a positive connection: model 1 (OR = 11.300, 95% CI: 7.034, 18.153), model 2 (OR = 8.714, 95% CI: 5.388, 14.091), and model 3 (OR = 2.776, 95% CI: 1.595, 4.831).

All BMI groups showed a positive correlation with the prevalence of NAFLD; additionally, those with a BMI <25 kg/m^2^ had a greater risk of developing NAFLD than those in other groups: model 1 (OR = 11.776, 95% CI: 5.762, 24.067), model 2 (OR = 6.629, 95% CI: 3.048, 14.417), and model 3 (OR = 5.383, 95% CI: 1.974, 4.680).

### Correlation between RC/HDL-C and the severity of hepatic steatosis

Supplementary Table 1 shows the relationship between RC/HDL-C and hepatic steatosis depending on CAP levels. In model 1 (β = 67.737, 95% CI: 60.855, 74.620), model 2 (β = 58.834, 95% CI: 51.881, 65.786), and model 3 (β = 21.513, 95% CI: 14.847, 28.178), it was demonstrated that RC/HDL was significantly and positively correlated with the degree of hepatic steatosis, whereas the *P* for trend was <0.001. Furthermore, compared to the lowest quartile, the higher RC/HDL quartile showed a substantially greater degree of hepatic steatosis (*P* for trend <0.001). After correcting for all pertinent variables, the positive correlation between RC/HDL-C and degree of hepatic steatosis persisted in males (β = 17.463, 95% CI: 8.643, 26.283, *P* < 0.001) and females (β = 26.630, 95% CI: 16.070, 37.189, *P* < 0.001), respectively. Simultaneously, the degree of hepatic steatosis was strongly linked with the RC/HDL-C in the BMI subgroup analysis, particularly in the group with a BMI of <25 kg/m^2^.

### Association between RC/HDL-C and the severity of liver fibrosis

We also investigated the connection between the three stages of hepatic fibrosis and RC/HDL-C. Our findings showed that in models 1 and 2, RC/HDL-C was significantly associated with either significant fibrosis, advanced fibrosis, or cirrhosis. However, RC/HDL-C was not observed to be associated with liver fibrosis in model 3, even after accounting for all potential confounders (significant fibrosis: OR = 1.113, 95% CI: 0.710, 1.743; advanced fibrosis: OR = 1.217, 95% CI: 0.707, 2.096; cirrhosis: OR = 1.401, 95% CI: 0.679, 2.891), as presented in Supplementary Table 2. Furthermore, we discovered in the gender subgroup analysis that there was no association between liver fibrosis and RC/HDL-C level after correcting all pertinent covariables.

### The analysis of non-linear relationship

**Figure 2:**
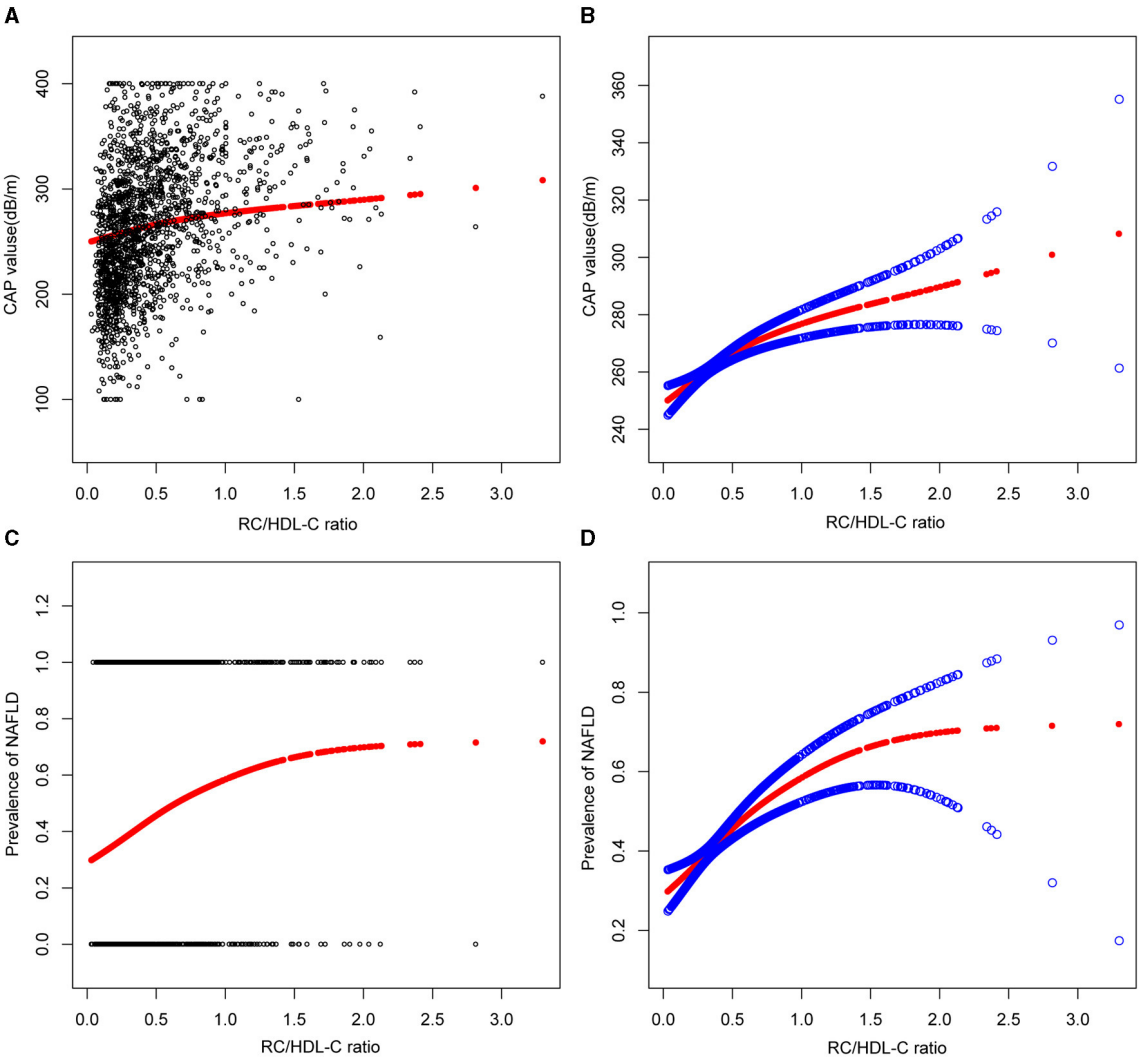
Associations between remnant cholesterol to high-density lipoprotein cholesterol ratio and CAP values or prevalence of NAFLD. **(A, B)** RC/HDL-C ratio and CAP values Associations. **(C, D)** Associations between RC/HDL-C ratio and prevalence of NAFLD. Each black point represents a sample. The solid red line represents the smooth curve fit between variables. Blue bands represent the 95% confidence interval from the fit.

**Figure 3:**
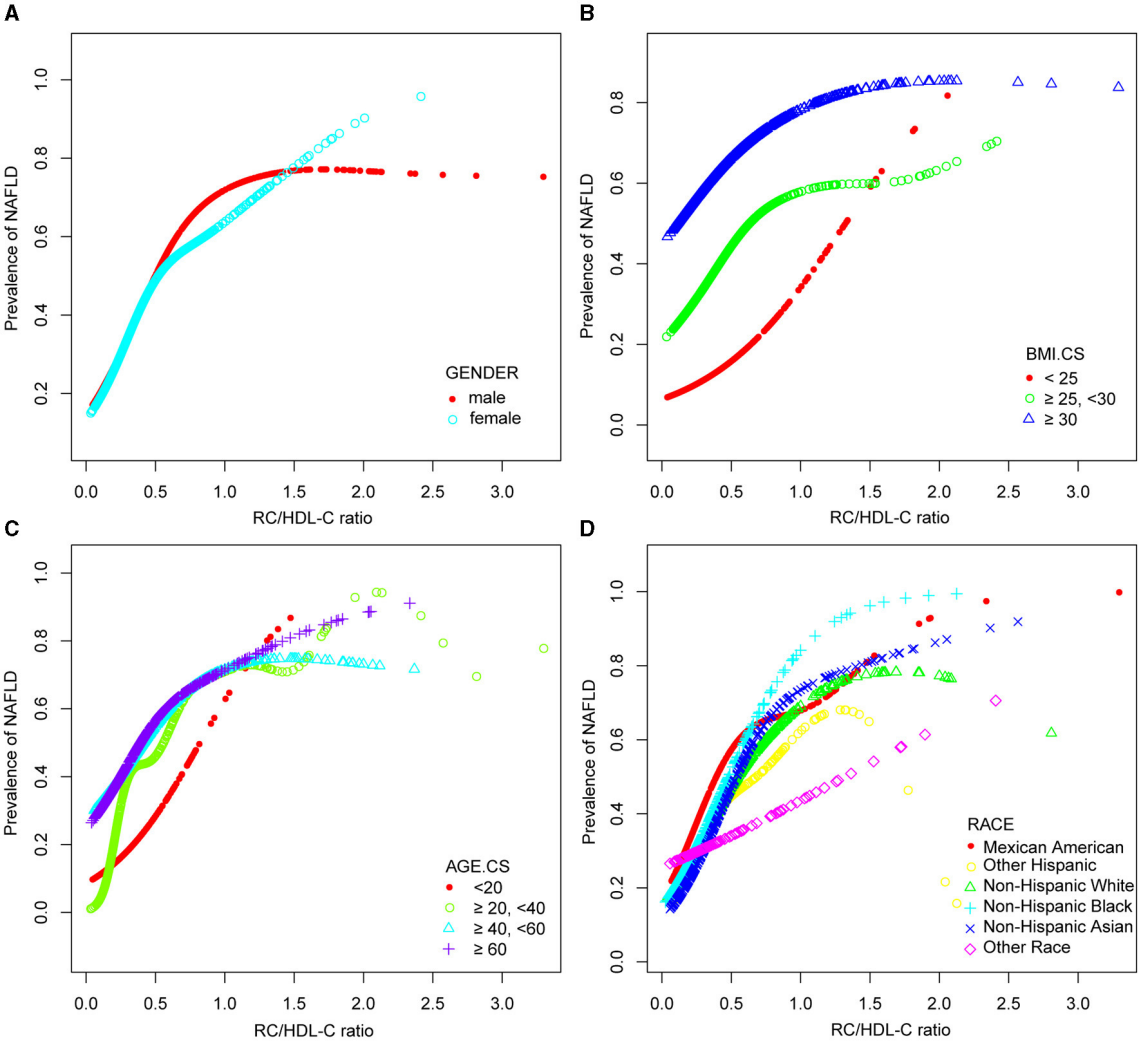
Associations between remnant cholesterol to high-density lipoprotein cholesterol ratio and the prevalence of NAFLD by gender **(A)**, BMI **(B)**, age **(C)**, and race **(D)**. They were adjusted for age, gender, race, hypertension, BMI, T2DM, smoke, LSM, DBP, SBP, HBA1C, CRP, PLT, fast glucose, fast insulin, HbA1c, ALT, AST, GGT, TC, TBIL, and SUA. In subgroup analyses, the model was not adjusted for the classified variables.

In the context of our current investigation, we investigated the potential for a non-linear relationship between RC/HDL-C and NAFLD using smooth curve fits. Subgroup analyses used age, race, gender, and BMI categories. The correlation between RC/HDL-C and the incidence of NAFLD showed a non-linear pattern, as shown in Table 3, Figures 2, 3. The inflection point for all participants was 0.619, 0.690 for the male, 0.617 for the BMI 25–30 kg/m^2^ subgroup population, and 1.257 for the cohort aged 20–40 years.

**Table 3:** Threshold effect analysis of RC/HDL-C on the prevalence of NAFLD in different genders and ages using the two-piecewise linear regression model.

**Prevalence of NAFLD**	**Adjusted OR (95% CI), *P* value**
**All participants**	
Fitting by the standard linear model	2.734 (1.895, 3.944), <0.0001
**Fitting by the two-piecewise linear model**	
Inflection point	0.619
RC/HDL-C <0.619	6.484 (2.932, 14.341), <0.0001
RC/HDL-C > 0.619	1.568 (0.898, 2.737), 0.1137
Log likelihood ratio	0.016
**Men**	
Fitting by the standard linear model	2.613 (1.582, 4.316), 0.0002
**Fitting by the two-piecewise linear model**	
Inflection point	0.690
RC/HDL-C <0.690	11.398 (3.703, 35.079), <0.0001
RC/HDL-C > 0.690	1.032 (0.488, 2.182), 0.9351
Log likelihood ratio	0.004
**AGE 20–40 (years)**	
Fitting by the standard linear model	4.689 (2.206, 9.968), <0.0001
**Fitting by the two-piecewise linear model**	
Inflection point	1.257
RC/HDL-C <1.257	10.049 (3.902, 25.880), <0.0001
RC/HDL-C > 1.257	0.304 (0.053, 1.732), 0.1797
Log likelihood ratio	0.010
**BMI 25–30 (kg/m** ^2^ **)**	
Fitting by the standard linear model	2.944 (1.601, 5.416), 0.0005
**Fitting by the two-piecewise linear model**	
Inflection point	0.617
RC/HDL-C <0.617	17.962 (4.641, 69.526), <0.0001
RC/HDL-C > 0.617	1.016 (0.422, 2.448), 0.9712
Log likelihood ratio	0.003

Age, gender, race, hypertension, BMI, T2DM, smoke, LSM, DBP, SBP, HBA1C, CRP, PLT, fast glucose, fast insulin, ALT, AST, GGT, TC, TBIL, and SUA were adjusted. In the subgroup analysis for sex, the model was not adjusted for sex. In the subgroup analysis for age, the model was not adjusted for age; in the subgroup analysis for BMI, the model was not adjusted for BMI.

### RC/HDL-C as a predictor of NAFLD: ROC analysis

**Figure 4:**
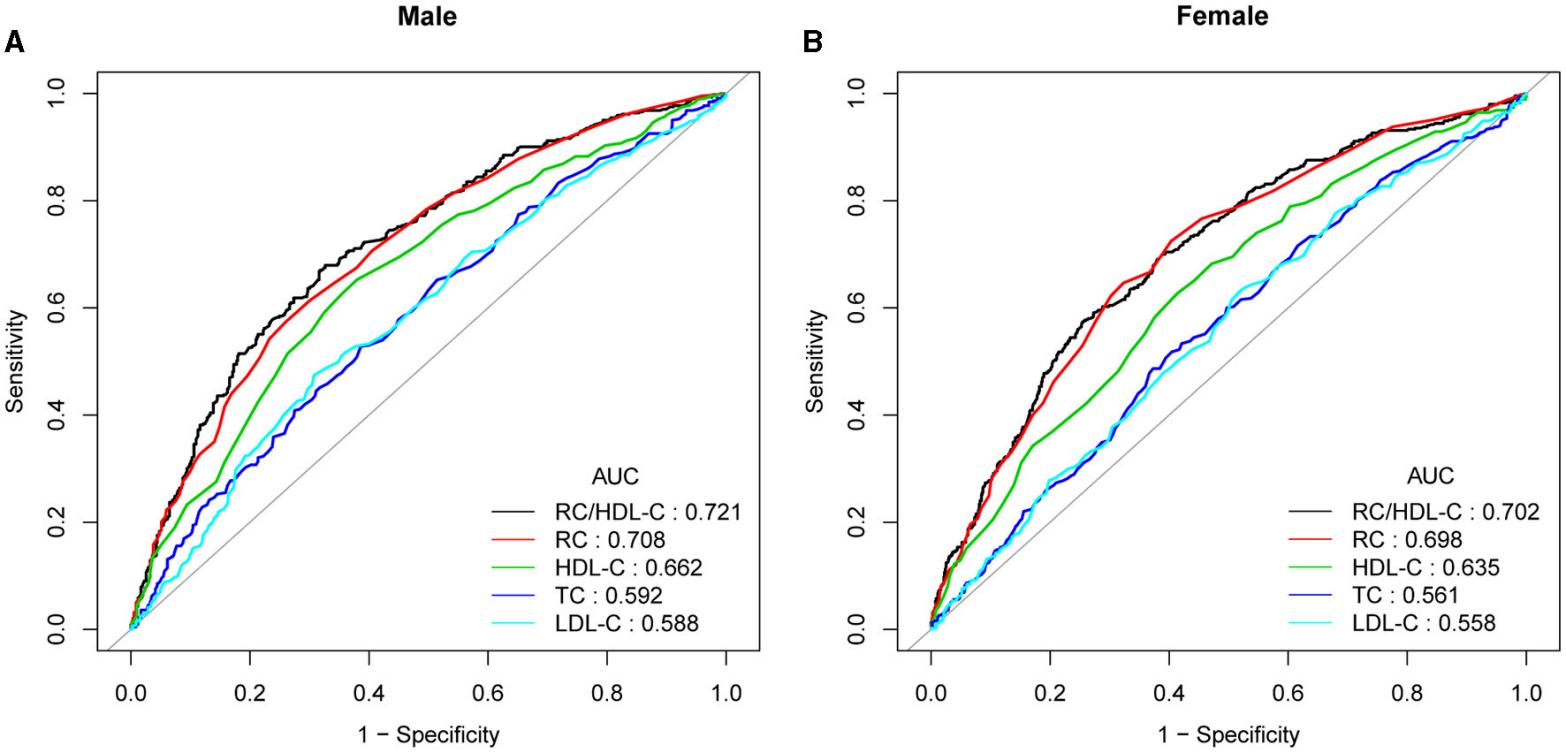
ROC curves for RC/HDL-C, compared to RC, LDL-C, TC, and HDL-C for NAFLD onset among males **(A)** and females **(B)**. The predictive value for RC/HDL-C is more significant than those other factors, as determined by its AUC.

In previous research, ROC curve analysis identified HDL-C, LDL-C, RC, and TC as significant predictors of NAFLD. Figure 4, Supplementary Figure 1, Supplementary Table 3 displayed the ROC for RC/HDL-C compared to these predictors. As shown in Supplementary Table 3, the ROC analysis's area under the curve (AUC) for RC/HDL-C was 0.7139 (95% CI: 0.6923, 0.7354), substantially more significant compared to that of HDL-C, LDL-C, RC, and TC (*P* < 0.001). The estimated RC/HDL-C's sensitivity and specificity of NAFLD were 71.58% and 62.26%, respectively. We further conducted a subgroup analysis based on gender and found that AUC for RC/HDL-C was 0.7211 (95% CI: 0.6968, 0.7523) in males and 0.7022 (95% CI: 0.6721, 0.7323) in female, which revealed that the AUC for RC/HDL-C in the ROC analysis exceeded that of the other indicators, as demonstrated in Figure 4, Supplementary Table 3.

## Discussion

The current national cross-sectional study examined the association between the RC/HDL-C ratio and incident NAFLD in US populations. The results demonstrate that a high RC/HDL-C ratio is independently and robustly correlated with an increased risk of NAFLD and hepatic steatosis events. In brief, the results primarily demonstrated that the individuals in the NAFLD group had a significantly higher RC/HDL-C level than those in the non-NAFLD group. Remarkably, we found that a unit increase in RC/HDL-C was linked to a 2.734-fold increase in the incidence of NAFLD, even after correcting for all relevant factors. Notably, subgroup analysis based on gender and BMI categories revealed statistically significant positive relationships. Furthermore, the study demonstrated a strong correlation between RC/HDL-C and hepatic steatosis. In addition, the ROC analysis revealed that RC/HDL-C was more effective in identifying NAFLD than either RC or HDL-C. The RC/HDL-C ratio is a relatively simple indicator to compute and has the potential to be a valuable future predictor of the risk of NAFLD in the general population.

Previous research has concentrated on the connection between cerebral atherosclerotic stenosis, coronary heart disease development, and perioperative myocardial injury with the RC/HDL-C ratio (11, 13). Simultaneously, research has demonstrated that the RC/HDL-C ratio is the most accurate indicator of diabetes risk, and it also shows a higher predictive value for prediabetes than conventional lipid measures (20, 21). Nevertheless, the relationship between the RC/HDL-C ratio and NAFLD has not been extensively investigated. A recent study by Zou et al. examined 14,251 individuals in Japan using a series of cross-sectional investigations based on a secondary analysis of NAGALA cohort data. The study also conducted a mediating analysis to investigate the function of lipid parameters in the relationship between NAFLD and BMI. The results demonstrated that the RC/HDL-C ratio played a more significant role in mediating this association than traditional lipid parameters. Additionally, the diagnostic value of each lipid parameter was found to be inferior to that of the ratio of the two lipid parameters for NAFLD (22). Concurrently, based on the NAGALA database, Zou et al. found that the strongest indication for identifying NAFLD in the adult cohort was a high RC/HDL-C ratio, with an AUC of 0.82 and an ideal cutoff value of 0.43, which enhanced the diagnostic potential of the disease (23, 24). Our study's conclusion is in line with earlier research findings. The connection between RC/HDL-C and NAFLD is strong, and compared to other conventional lipid markers, the AUC of RC/HDL-C for NAFLD is substantially greater. Nevertheless, in previous studies, the subjects were all Asian. In this investigation, the risk of NAFLD and RC/HDL-C in a sizable sample of the US general population is being examined for the first time. Based on these findings, it appears that the RC/HDLC ratio may be a useful metabolic indicator for the diagnosis of NAFLD in Asians as well as other ethnic groups in the US. Further research is required to elucidate the relationship between these two factors.

The pathophysiology of NAFLD is complex and may involve multiple mechanisms. Firstly, dyslipidemia has been demonstrated to significantly influence the development of NAFLD. Furthermore, non-traditional lipids are more helpful in diagnosing metabolic disorders than traditional lipid parameters. The potential mechanism underlying the correlation between RC and NAFLD remains unclear, with several studies proposing explanations for the relationship between RC and NAFLD (25). RC is a TG-rich cholesterol. An increase in RC level leads to the accumulation of lipids in the liver, which results in an imbalance of cholesterol homeostasis in this organ, which, in turn, produces lipotoxicity, causes oxidative stress, an increase of reactive oxygen species, and ultimately leads to mitochondrial dysfunction (26, 27). Secondly, there is evidence from multiple studies that elevated levels of RC are associated with low-grade inflammation and may be linked to tumor necrosis factor, interleukin-1, 6, and 8, as well as adhesion molecules that facilitate atherosclerosis (28, 29). Oxidative stress and inflammation, which play a significant role in the pathophysiology of NAFLD, might contribute to the association between NAFLD and RC (30). The following factors are connected to HDL-C's involvement in the onset and progression of NAFLD. In addition to its participation in cholesterol transport, HDL-C can transfer cholesterol from extrahepatic tissue to the liver for metabolic processes (31, 32). Given the pivotal role that systemic low-grade inflammation plays in the pathogenesis of NAFLD, HDL-C exhibits noteworthy anti-inflammatory properties by inhibiting pro-inflammatory cytokines and chemokines and reducing adhesion molecule expression (10, 33). Furthermore, there is a negative correlation between insulin resistance and HDL diameter. Consequently, a decrease in serum HDL-C levels leads to a decline in cholesterol clearance, an increase in pro-inflammatory factors, and enhancing insulin resistance, which is conducive to the advancement of NAFLD. Consequently, it is speculated that the RC/HDLC ratio may be tightly linked to inflammation, oxidative stress, IR, and aberrant lipid metabolism (34).

Furthermore, we analyzed the non-linear association between NAFLD prevalence and RC/HDL-C, as well as the possibility of a saturation effect of RC/HDL-C in predicting NAFLD risk. To date, no similar research results have been reported in the literature. The study's findings indicated an inverted U-shaped pattern connection between the prevalence of NAFLD and men's RC/HDL-C, with an inflection point of 0.69. Consequently, it is recommended that the function of RC/HDL-C in different genders be considered when estimating the risk of NAFLD. The findings may provide new insights for the prevention and treatment of NAFLD.

Furthermore, our study revealed a significant association between the degree of hepatic steatosis and the RC/HDL-C ratio in the US NAFLD population. This finding is of particular interest, as it builds upon previous research that has demonstrated a correlation between RC, liver fibrosis, and hepatic steatosis (35–43). A cross-sectional study of 6,053 healthy participants who underwent a physical examination in China revealed a positive correlation between serum RC levels and CAP values, irrespective of conventional lipid parameters. Furthermore, the higher the serum RC level, the more severe liver steatosis (35). Moreover, Chin et al. identified a significant correlation between the degree of hepatic steatosis and the serum RC level (36). However, no studies have reported the relationship between RC/HDL-C levels and the severity of liver steatosis and fibrosis. Our study, which was based on CAP values, demonstrated a strong correlation between the degree of hepatic steatosis and RC/HDL-C levels. It is therefore suggested that RC/HDL-C levels may be beneficial biomarkers for predicting the treatment of hepatic steatosis in patients with NAFLD. A recent study by Wang et al. found that serum RC in patients with NAFLD was positively correlated with liver fibrosis. Furthermore, it was demonstrated that serum RC was a more robust indicator of liver fibrosis diagnosis than other lipid parameters (37). Our research did not identify a statistically significant correlation between the RC/HDL-C level and the LSM value. This may be attributed to the limited sample size and the fact that LSM is not the gold standard for identifying NAFLD fibrosis. Further research is required to investigate the mechanisms between them.

The following are the significant strengths of our research. First, the most crucial aspect of the study is the large population size, and the fact that the population studied is representative of the general population of the United States. Second, based on TE detection, this research demonstrates for the first time that the RC/HDL-C ratio may predict the degree of hepatic steatosis independently. However, it should be noted that this study has certain limitations. Firstly, as this is a cross-sectional observational study, it is not possible to establish a causal connection between NAFLD and RC/HDL-C. Therefore, further prospective research is required to clarify the connection and mechanism between RC/HDL-C and NAFLD. Secondly, TE with a CAP value of ≥ 274 dB/m is employed to diagnose NAFLD instead of liver biopsy, which may result in discrepancies in the diagnosis of NAFLD patients. The most reliable method for diagnosing NAFLD is a liver biopsy; liver pathology can provide further histological details, such as liver fibrosis and inflammation. Thirdly, the findings of RC calculations may differ since they are based on standard biochemical data rather than precise measurements. Fourthly, even after adjusting for several significant covariates, there is the possibility that other variables, such as hypertension and diabetes treatment medications, might still be confounding factors. Future research should address these difficulties.

## Conclusion

In conclusion, a higher RC/HDL-C ratio in the US population was independently associated with a significantly higher risk of developing NAFLD and an increased degree of hepatic steatosis. RC/HDLC ratio can be used as a simple and effective non-invasive biomarker to identify individuals with high risk of NAFLD, which is helpful for the early detection of this prevalent liver disease.

## Data availability statement

The datasets presented in this study can be found in online repositories. The names of the repository/repositories and accession number(s) can be found below: https://wwwn.cdc.gov/nchs/nhanes/continuousnhanes/default.aspx?BeginYear=2017.

## Ethics statement

The studies involving humans were approved by the Ethics Review Board of the National Center for Health Statistics approved all NHANES protocols. The studies were conducted in accordance with the local legislation and institutional requirements. Written informed consent for participation was not required from the participants or the participants' legal guardians/next of kin in accordance with the national legislation and institutional requirements.

## Author contributions

YX: Conceptualization, Investigation, Writing – original draft, Writing – review & editing, Data curation, Formal analysis, Software. WH: Writing – original draft, Conceptualization, Data curation. YW: Investigation, Software, Writing – original draft. JL: Methodology, Writing – original draft. LY: Software, Writing – original draft. SY: Writing – review & editing. DZ: Writing – review & editing.
