# Supplementary material for: Association between RC/HDL-C ratio and risk of non-alcoholic fatty liver disease in the United States
Source: Front Med (Lausanne). 2024 Jul 29;11:1427138. doi: 10.3389/fmed.2024.1427138 (PMC11317378; doi:10.3389/fmed.2024.1427138)
Supplement: Supplementary file 1 [file Data_Sheet_1.docx]

Supplementary Material

**1 Supplementary Figures and Tables**

**1.1 Supplementary Figures**

**
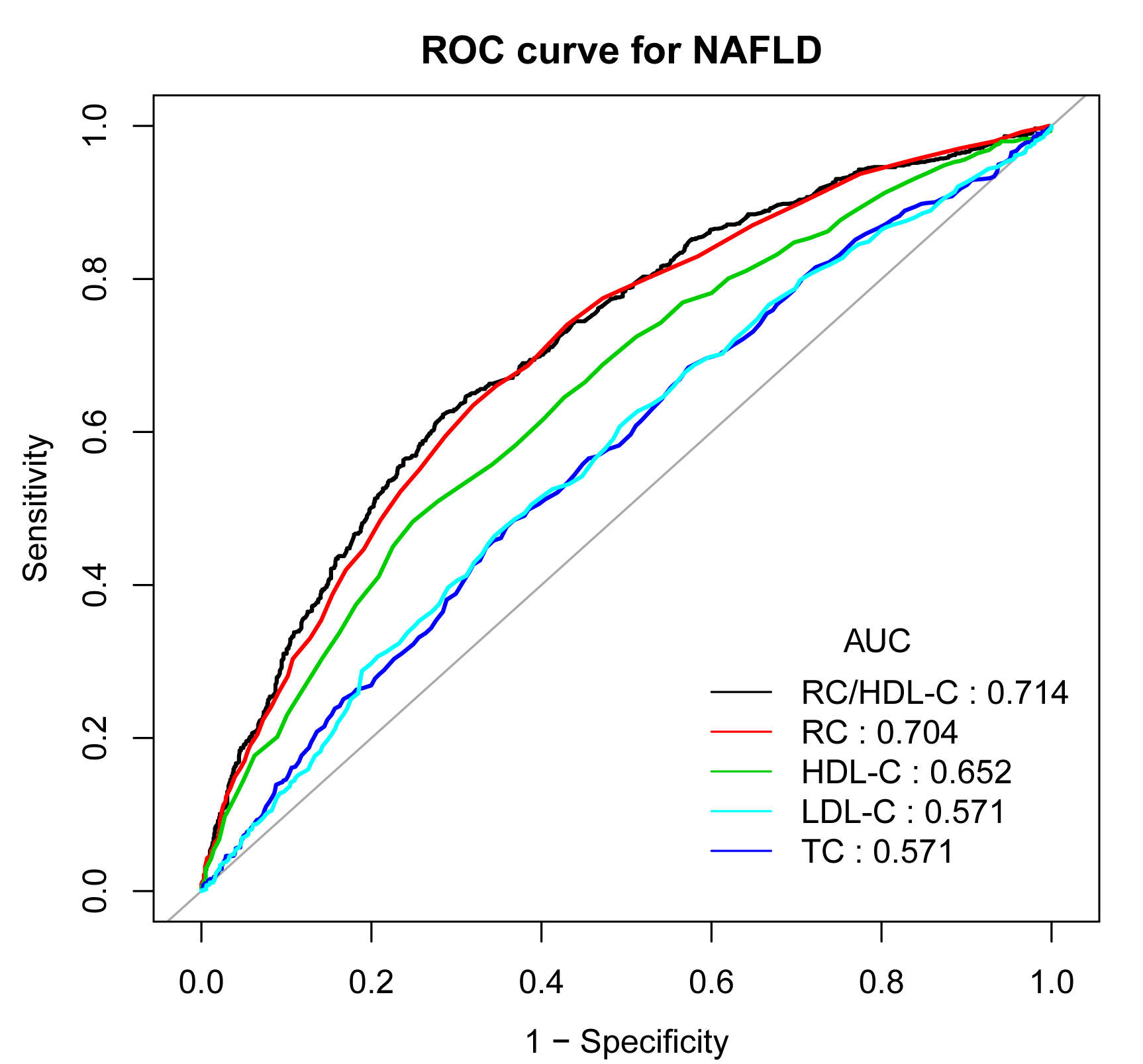
**

**Supplemental Figure 1.** ROC curves for RC/HDL-C, compared to RC, LDL-C, TC, and HDL-C for NAFLD onset. The predictive value for RC/HDL-C is more significant than other factors, as determined by AUC.

**1.2 Supplementary Tables**

**Supplemental** **Table 1**. Correlation between RC/HDL-C and hepatic steatosis based on CAP value.

|  | **Model 1 β (95% CI), *P* value** | **Model 2 β (95% CI), *P* value** | **Model 3 β (95% CI), *P* value** |
| --- | --- | --- | --- |
| RC/HDL-C  (per SD increase) | 67.737 (60.855, 74.620) < 0.001 | 58.834 (51.881, 65.786)  < 0.001 | 21.513 (14.847, 28.178)  < 0.001 |
| Q1 (0.303-1.282) | Reference | Reference | Reference |
| Q2 (1.283-2.075) | 22.978 (16.199, 29.758)  < 0.001 | 20.075 (13.438, 26.713)  < 0.001 | 4.794 (-1.774, 11.362)  0.152 |
| Q3 (2.077-3.204) | 44.337 (37.560, 51.114) < 0.001 | 36.491 (29.696, 43.287)  < 0.001 | 11.684 (4.835, 18.533)  < 0.001 |
| Q4 (3.205-9.951) | 71.066 (64.295, 77.836)  < 0.001 | 61.872 (54.909, 68.834)  < 0.001 | 21.283 (14.040, 28.527)  < 0.001 |
| *P* for trend | < 0.001 | < 0.001 | < 0.001 |
| **Subgroup analysis stratified by sex** | | | |
| Men 60.298 (51.039, 69.558) 52.249 (43.034, 61.464) 17.463 (8.643, 26.283)  < 0.001 < 0.001 < 0.001 | | | |
| Women 76.980 (66.253, 87.707) 68.628 (57.800, 79.457) 26.630 (16.070, 37.189)  < 0.001 < 0.001 < 0.001 | | | |
| **Subgroup analysis stratified by BMI** | | | |
| <25 | 63.297 (50.808, 75.785)  < 0.001 | 46.224 (33.647, 58.801)  < 0.001 | 33.725 (18.998, 48.452)  < 0.001 |
| ≥ 25, <30 | 41.575 (30.148, 53.002)  < 0.001 | 33.515 (21.912, 45.118)  < 0.001 | 21.411 (9.040, 33.782)  < 0.001 |
| ≥ 30 | 40.553 (30.767, 50.338) < 0.001 | 30.171 (20.074, 40.268) < 0.001 | 16.269 (6.255, 26.283)  0.001 |

Model 1: no covariates were adjusted. Model 2: age, gender, and race were adjusted. Model 3:age, gender, race, hypertension, BMI, T2DM, smoke, LSM, DBP, SBP, HBA1C, CRP, PLT, fast glucose, fast insulin, HbA1c, ALT, AST, GGT, TC, TBIL, and SUA were adjusted. In the subgroup analysis for sex, the model was not adjusted for sex, and in the subgroup analysis for BMI, the model was not adjusted for BMI.

**Supplemental** **Table 2.** Association between RC/HDL-C and risk of hepatic fibrosis.

| **Degree of liver fibrosis** | **Model 1 *OR* (95% CI), *P* value** | **Model 2 *OR*(95% CI), *P* value** | **Model 3 *OR* (95% CI), *P* value** |
| --- | --- | --- | --- |
| Significant fibrosis (F2, LSM ≥ 8.0) | 1.808 (1.285, 2.546) < 0.001 | 1.731 (1.204, 2.488) 0.003 | 1.113 (0.710, 1.743) 0.641 |
| Advanced fibrosis (F3, LSM ≥ 9.7) | 1.911 (1.272, 2.872) 0.001 | 1.701 (1.096, 2.642) 0.017 | 1.217 (0.707, 2.096) 0.479 |
| Cirrhosis  (F4, LSM ≥ 13.7) | 2.007 (1.162, 3.467) 0.012 | 1.807 (1.001, 3.264) 0.049 | 1.401 (0.679, 2.891) 0.361 |
| **Subgroup analysis stratified by gender** | | | |
| **Men** |  |  |  |
| Significant fibrosis (F2, LSM ≥ 8.0) | 1.545 (1.001, 2.384) 0.049 | 1.506 (0.956, 2.372) 0.077 | 0.948 (0.538, 1.669) 0.853 |
| Advanced fibrosis (F3, LSM ≥ 9.7) | 1.880 (1.146, 3.082) 0.012 | 1.731 (1.024, 2.928) 0.040 | 1.178 (0.613, 2.264) 0.622 |
| Cirrhosis  (F4, LSM ≥ 13.7) | 2.211 (1.199, 4.079) 0.011 | 2.022 (1.047, 3.904) 0.036 | 1.751 (0.774, 3.961) 0.178 |
| **Women** |  |  |  |
| Significant fibrosis (F2, LSM ≥ 8.0) | 2.425 (1.380, 4.262) 0.002 | 2.294 (1.233, 4.270) 0.008 | 1.382 (0.618, 3.093) 0.431 |
| Advanced fibrosis (F3, LSM ≥ 9.7) | 1.982 (0.965, 4.067) 0.062 | 1.614 (0.719, 3.622) 0.245 | 1.026 (0.353, 2.980) 0.963 |
| Cirrhosis  (F4, LSM ≥ 13.7) | 1.457 (0.443, 4.792) 0.535 | 1.093 (0.278, 4.294) 0.899 | 0.604 (0.096, 3.795) 0.591 |

Model 1: no covariates were adjusted. Model 2: age, gender, and race were adjusted. Model 3:age, gender, race, hypertension, BMI, T2DM, smoke, LSM, DBP, SBP, HBA1C, CRP, PLT, fast glucose, fast insulin, HbA1c, ALT, AST, GGT, TC, TBIL, and SUA were adjusted. In the subgroup analysis for sex, the model was not adjusted for sex.

**Supplemental** **Table 3**. The best threshold, sensitivities, specificities, and area under the curve of lipid-related parameters for each parameter in identifying NAFLD, and subgroup analysis for gender.

|  | **AUC** | **95% CI** | **Best threshold** | **Specificity** | **Sensitivity** |
| --- | --- | --- | --- | --- | --- |
| RC/HDL-C | 0.7139 | 0.6923-0.7354 | 0.3723 | 0.7158 | 0.6226 |
| RC | 0.7045 | 0.6828-0.7262 | 18.5000 | 0.6802 | 0.6349 |
| HDL-C | 0.6516 | 0.6286-0.6746 | 46.5000 | 0.7515 | 0.4826 |
| TC | 0.5709 | 0.5469-0.5950 | 184.5000 | 0.6323 | 0.4849 |
| LDL-C | 0.5715 | 0.5474-0.5955 | 112.5000 | 0.6555 | 0.4636 |
| **Subgroup analysis stratified by gender**  **Men** | | | | | |
| RC/HDL-C | 0.7211 | 0.6898-0.7523 | 0.3723 | 0.6735 | 0.6795 |
| RC | 0.7084 | 0.6768-0.7400 | 20.5000 | 0.7402 | 0.5734 |
| HDL-C | 0.6623 | 0.6289-0.6958 | 47.5000 | 0.6205 | 0.6524 |
| TC | 0.5916 | 0.5566-0.6266 | 176.5000 | 0.6137 | 0.5282 |
| LDL-C | 0.5883 | 0.5531-0.6234 | 113.5000 | 0.6923 | 0.4740 |
| **Women** |  |  |  |  |  |
| RC/HDL-C | 0.7022 | 0.6721-0.7323 | 0.3701 | 0.7459 | 0.5733 |
| RC | 0.6983 | 0.6682-0.7284 | 17.5000 | 0.6764 | 0.6467 |
| HDL-C | 0.6349 | 0.6029-0.6669 | 54.5000 | 0.5866 | 0.6267 |
| TC | 0.5611 | 0.5280-0.5942 | 187.5000 | 0.6271 | 0.4867 |
| LDL-C | 0.5582 | 0.5250-0.5913 | 98.5000 | 0.4829 | 0.6289 |

Abbreviations: AUC area under the curve; other abbreviations are in Table 1 and Table 2 .
